# ISRIB facilitates the co‐culture of human trophoblast stem cells and embryonic stem cells

**DOI:** 10.1111/cpr.13599

**Published:** 2024-01-12

**Authors:** Shuwei Xia, Dainan Yu, Yue Wang, Beijia He, Yin Rong, Shuo Chen, Zhenyu Xiao, Hongmei Wang, Hao Wu, Long Yan

**Affiliations:** ^1^ Key Laboratory of Organ Regeneration and Reconstruction, State Key Laboratory of Stem Cell and Reproductive Biology Institute of Zoology, Chinese Academy of Sciences Beijing China; ^2^ Institute for Stem Cell and Regeneration Chinese Academy of Sciences Beijing China; ^3^ Beijing Institute for Stem Cell and Regenerative Medicine Beijing China; ^4^ University of Chinese Academy of Sciences Beijing China; ^5^ School of Life Science Beijing Institute of Technology Beijing China

## Abstract

The embryo‐like structures (embryoids) constructed by aggregating embryonic stem cells (ESCs) and trophoblast stem cells (TSCs) have provided revolutionary tools for studying the intricate interaction between embryonic and extra‐embryonic tissues during early embryonic development, which has been achieved in mice. However, due to the opposite dependence on some signalling pathways for in vitro culture of human ESCs (hESCs) and TSCs (hTSCs), particularly WNT and TGFβ signalling pathways, which limits the construction of human post‐implantation embryoids by aggregating hESCs and hTSCs. To overcome this challenge, here, by screening 1639 chemicals, we found that an inhibitor of integrated stress response, ISRIB, can replace WNT agonists and TGFβ inhibitors to maintain the stemness and differentiation capacity of hTSCs. Thus, we developed an ISRIB‐dependent in vitro culture medium for hTSCs, namely nTSM. Furthermore, we demonstrated that ISRIB could also maintain the hESC stemness. Using a 3D co‐culture system (hESCs and hTSCs aggregate, ETA), we demonstrated that a 1:1 mixture of hESC culture medium (ESM) and nTSM improved the cell proliferation and organisation of both hESC‐ and hTSC‐compartments and the lumenogenesis of hESC‐compartment in ETAs. Overall, our study provided an ISRIB‐dependent system for co‐culturing hESCs and hTSCs, which facilitated the construction of human embryoids by aggregating hESCs and hTSCs.

## Graphical abstract

We identified ISRIB that could replace the chemicals targeting WNT and TGFβ signalling pathways in the current hTSC culture medium (TSM) by high‐throughput chemical screening. We developed a new ISRIB‐dependent hTSC culture medium, namely nTSM. We provided an ISRIB‐dependent strategy for co‐culturing of hESCs and hTSCs in vitro.

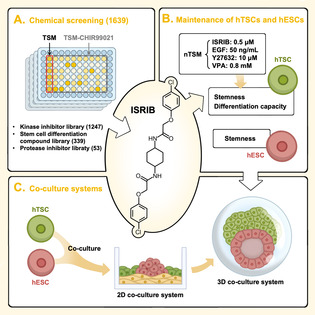

## 1 INTRODUCTION

Early human embryonic development depends on complex cell differentiation and spatial organisation and the support of extra‐embryonic tissues. At 7 days post fertilisation (dpf 7), the embryos undergo cell fate determination to form a blastocyst consisting of inner cell mass (ICM) and peripheral trophectoderm (TE).^1^ The ICM is composed of primitive endoderm (PrE) and epiblast (EPI). ICM will mainly develop into embryonic body, and PrE and TE will contribute to yolk sac and placenta, respectively, which belong to extra‐embryonic tissues.^2^, ^3^, ^4^ The intricate interactions between embryonic and extra‐embryonic tissues remain largely unknown. Recently, stem cell‐derived embryo‐like structures (embryoids) provide revolutionary tools for studying early embryonic development. In mice, assembling embryonic stem cells (ESCs) and trophoblast stem cells (TSCs) allowed the construction of gastrulating embryo‐like structures (gastruloids).^5^ However, unlike mice, it is inefficient to construct human embryoids by assembling hESCs and hTSCs owing to the lack of an appropriate co‐culture condition for hESCs and hTSCs.^6^, ^7^, ^8^, ^9^

Okae et al. firstly isolated hTSCs from human blastocysts and cytotrophoblast cells (CTBs) during early pregnancy by activating WNT and EGF and inhibiting TGFβ, HDAC and ROCK.^10^ WNT signalling pathway is important for the development of TE during peri‐implantation stage and is highly enriched in the first‐trimester placentas.^11^ Activation of WNT signalling pathway is crucial for maintaining hTSCs in vitro.^10^, ^11^ Contrary to hTSCs, the activation of WNT disrupts the stemness of hESCs and induces the differentiation towards the extra‐embryonic mesoderm (EXMC) lineage.^12^ Besides, inhibition of TGFβ is also indispensable for the maintenance of hTSCs, whereas TGFβ is necessary for the maintenance of hESC stemness.^9^, ^10^, ^13^, ^14^ Thus, the in vitro maintenance of hESCs and hTSCs displays opposite dependence on WNT and TGFβ signalling pathways. A balanced culture condition with a uniform maintenance action on both hESCs and hTSCs is required for constructing human embryoids by assembling hESCs and hTSCs.

To overcome the opposing requirements of WNT and TGFβ signalling pathways for in vitro maintenance of hESCs and hTSCs, here, we first identified chemicals to replace WNT agonists for in vitro culture of hTSCs by high‐throughput (1639) chemical screening. We found a series of candidate chemicals, including the chemicals targeting the signalling pathways known to be important for hTSCs, such as FGFR2, VEGFR, TGFβ, PI3K and c‐MET. Notably, among them, we found that ISRIB, an inhibitor of integrated stress response, could replace both the WNT agonists and TGFβ inhibitors to maintain hTSC viability, stemness and differentiation capacity. Thus, we developed a new ISRIB‐dependent culture medium for in vitro culture of hTSCs, namely nTSM. Besides, we demonstrated that the WNT and TGFβ signalling pathways showed comparable expression levels in the hTSCs cultured in nTSM with those cultured in TSM. Furthermore, we demonstrated that the addition of ISRIB had no effect on the stemness of hESCs. Using a 3D co‐culture system for hESCs and hTSCs (hESCs and hTSCs aggregate, ETA), we found that a 1:1 mixture of nTSM and the hESC culture medium (ESM) promoted the cell proliferation, organisation and development of both hESC‐ and hTSC‐compartments in ETAs, and promoted the lumenogenesis of hESC‐compartment. In summary, we demonstrated that ISRIB could improve the maintenance of hESCs and hTSCs in the co‐culture system, which may help for constructing human embryoids to simulate the development of post‐implantation human embryos.

## 2 MATERIALS AND METHODS

### 2.1 Ethics statement

The study was approved by the Research Ethics Committee (Research licence 2019SZZX‐008) of the Sixth Affiliated Hospital of Sun Yat‐Sen University.

### 2.2 Cell culture

The hTSC lines used in this study included the hTSCs established by Wang lab and the bTS11 gifted by Arima lab,^10^, ^15^ and the culture and differentiation of hTSCs were performed following our previously published protocol.^16^ Briefly, the hTSCs were cultured in the TSM, which was prepared as DMEM/F12 (Gibco, #11320033) supplemented with 0.1 mM 2‐mercaptoethanol (Gibco, #21985023), 0.2% FBS (Gibco, #10091), 0.5% Penicillin–Streptomycin (Gibco, #15140122), 0.3% BSA (MP Biomedicals, #0218072801), 1% ITS‐X supplement (Gibco, #51500056), 1.5 μg/mL l‐ascorbic acid (Sigma‐Aldrich, #33034), 50 ng/mL EGF (MCE, #HY‐P7109), 2 μM CHIR99021 (MCE, #HY‐10182), 0.5 μM A83‐01 (MCE, #HY‐10432), 1 μM SB431542 (MCE, #HY‐10431), 0.8 mM VPA (Wako, #227‐01071) and 5 μM Y27632 (MCE, #HY‐10071).

The new TSM (nTSM) established in this study was prepared as DMEM/F12, 0.1 mM 2‐mercaptoethanol, 0.2% FBS, 0.5% Penicillin–Streptomycin, 0.3% BSA, 1% ITS‐X supplement, 1.5 μg/mL L‐ascorbic acid, 50 ng/mL EGF, 0.8 mM VPA, 10 μM Y27632 and 0.5 μM ISRIB (TargetMol, #T6183).

The hTSCs were passaged every 4–5 days at a 1:6 split ratio. For passaging, hTSC colonies were dissociated to single cells by incubation with TrypLE (Gibco, #12604013) at 37°C for 8 min. For STB differentiation, the hTSCs at single‐cell state were seeded in a six‐well plate at 1 × 10^5^ cells per well and were cultured in the STB differentiation medium, which was prepared as DMEM/F12 medium supplemented with 0.3% BSA, 1% ITS‐X, 0.5% penicillin–streptomycin and 0.1 mM 2‐Mercaptoethanol, 2.5 μM Y27632, 4% KSR and 2 μM Forskolin.

The hESCs (RUES2) used in this study were kindly gifted by Brivanlou lab and were cultured in Essential 8 Medium (E8, Gibco, #A1516901) or mTeSR Plus medium (STEMCELL, #100‐0276). For passaging, hESC colonies were dissociated into 8–12 cell clumps by incubation with 0.5 mM EDTA (Invitrogen™, # 15575020) at 37°C for 10 min. Then, they were passaged at a 1:20 split ratio. For obtaining hESC single cells, the hESC colonies were incubated with Accutase (Invitrogen, A1110501) at 37°C for 8 min, and the cells were isolated by centrifugation at 1200 rpm for 3 min.

We established hTSC‐GFP cell line using lentivirus‐plasmids carrying GFP‐Puro, which was performed following our previously published protocol.^17^ The hESC‐RFP cell line was established using lentivirus‐plasmids carrying RFP‐Puro as well.

### 2.3 Minus one experiment

The hTSCs were cultured in the TSM removed CHIR99021, VPA, A83‐01, Y27632 and EGF, respectively. Then, the cells were analysed after a 4‐day culture. The above strategy was referred to minus one experiment. We used minus one experiment to evaluate the role of each supplement in current TSM on hTSC maintenance.

### 2.4 High‐throughput chemical screening

The cell viability was measured by Cell Counting Kit‐8 (CCK‐8, LABLEAD, #CK001‐500T) according to the manufacturer's instruction: 10 μL of CCK‐8 was added to each well and incubated at 37°C for 2 h. At the first‐round screening, 2000 hTSCs (established in our lab)^15^ were seeded into each well of 96‐well plates. The cells were treated with 1639 compounds, respectively (the final concentration of 1 μM) in the CHIR99021‐free culture system. The compound library consists of 339 stem cell differentiation compounds, 1247 kinase inhibitors and 53 protease inhibitors. Three replicate wells were set up for each compound. After 4 days of treatment, the cell viability was measured using a microplate reader (BioTek, Agilent) set to 450 nm. After minus the absorbance of the cell culture medium, we selected the compounds with the maximum absorbance for the next round of screening. We also performed the minus‐one experiment for the second‐round screening, using hTSC line established by our lab and bTS11 from Arima lab.^10^

### 2.5 2D co‐culture system

We seeded hESCs and hTSCs in equal proportions on feeder cells. Feeder cells need to be seeded 1 day in advance. hESCs and hTSCs were added into ESM, TSM, ESM + TSM, ESM + TSM + ISRIB, ESM + TSM‐3C, ESM + TSM‐3C + ISRIB medium at the same time, and the medium was changed half a day. Morphological observations were performed daily. On the fourth day, all cells were collected by TryPLE digestion and flow assayed. Feeder cells were removed by adsorption with 1% gelatin plates.

### 2.6 3D co‐culture system

The generation of hESCs and hTSCs aggregates (ETA) was performed following our previously published protocol.^18^ The AggreWell™ 400 plates were rinsed with 1 mL rinsing solution (Stem Cell Technologies, 07010) per well. Then, the plates were centrifuged for 5 min at 2000*g* and incubated with rinsing solution at room temperature for 20 min. After incubation, the wells were washed with 2 mL of 1× DPBS (Dulbecco's Phosphate Buffered Saline). After that, 500 μL ESM containing 10 μM Y27632 was added to the AggreWell and incubated at 37°C for use. On Day −1, 14,400 hESCs were seeded into AggreWell™ 400 plate per well. The hESCs were cultured in 1.5 mL ESM supplemented with 10 μM Y27632 per well. On Day 0, 500 μL ESM was removed, and 80,000 hTSCs at single‐cell state were seeded per well. The ETAs were cultured in a 1:1 mixture of ESM and the indicated mediums, including ESM, TSM, TSM‐3C (TSM removed A83‐01, SB431542 and CHIR99021) and nTSM. And the final concentration of Y27632 was adjusted to 10 μM. Then, the co‐culture system was incubated at 37°C and 5% CO_2_ for the following days. On Day 3, the ETAs were transferred to low‐adherence plate, and were cultured in the APEL2 medium supplemented with 3 μM CHIR99021, 50 ng/mL EGF and 40% Matrigel (Corning, 354230). On Day 4, the medium was replaced with the APEL2 supplemented with 3 μM CHIR99021, 5 ng/mL FGF2 and 50 ng/mL EGF.

### 2.7 Quantitative real‐time PCR analysis

Total RNA was extracted with the TRIzol reagent (Invitrogen, #15596018). cDNA was synthesised using a reverse transcription kit (Vazyme, #R333‐01) following the manufacturer's protocol. A list of gene‐specific primers used can be found in Table S1. Quantitative real‐time PCR (qRT‐PCR) was performed using TB Green Fast qRT‐PCR Mix (Takara, #RR430A) on a Roche Light Cycler 480 System. The amount of target mRNA was determined using the 2^−ΔΔCT^ method with GAPDH as the internal control.

### 2.8 Immunofluorescence staining

Cells were fixed using 4% PFA and permeated by 0.5% Triton X‐100. After blocking with 3% BSA solution for 60 min, cells were stained with primary antibodies overnight at 4°C. Antibodies to OCT4 (1:200; Santa Cruz Biotechnology), GATA3 (1:200; Abcam), TEAD4 (1:200; Cell Signalling Technology), KI67 (1:200; Cell Signalling Technology) and F‐actin/Phalloidin (1;500; YEASEN) were used in this study. Cells were then incubated for 1 h at room temperature with Alexa Fluor 488 green‐fluorescent or Alexa Fluor 568 red‐fluorescent (Invitrogen) at 1:200. DAPI was used to label cell nuclei. Images were acquired using Leica Stellaris, Andor Dragfly 200 and Nikon Ti2 microscopes. Images were analysed using Leica LAS X, Imaris 6.0 and Nikon NIS software.

### 2.9 Flow cytometry

Cells were dissociated with TrypLE into single cells and washed three times with DPBS. After filtering with a 40‐μm cell strainer (Falcon, #352340), samples were run on a BD Fortessa System (BD Sciences, Franklin Lakes, NJ, USA). Data were analysed with FlowJo software. All experiments were performed in three replicates.

### 2.10 RNA‐sequencing

Total RNA was extracted with the TRIzol reagent and used for library construction. The libraries were sequenced on the Illumina Novaseq 6000 platform (Illumina). The reads were aligned to the reference genome (GRCh38.p7) using Hisat 2.0 with the Refseq gene annotation. Expression levels (Count) of Refseq genes were calculated using StringTie 1.3.0. Differentially expressed genes were identified using Ballgown, and genes with *p* < 0.05 were considered as differentially expressed genes. Gene lists for pre‐CTB, post‐CTB, early‐STB, STB, early‐EVT and EVT were obtained from the data published by Ohgushi et al.^19^ (Table S2). Kyoto Encyclopedia of Genes and Genomes (KEGG) pathway was performed using cluster Profiler packages in R platform, and *p* < 0.05 was set as the threshold. All heat maps were performed using ggplot2 and pheatmap packages in the R environment (v 4.1.0). Enrichment bar charts were constructed with the export, readxl, ggplot2 and export packages.

### 2.11 Statistical analysis

GraphPad Prism 9 was used for statistical analyses and graph plotting. Student's *t* tests were performed to calculate statistical significance. *, *p* < 0.05. **, *p* < 0.01. ***, *p* < 0.001. ****, *p* < 0.0001. ns, no significance.

## 3 RESULTS

### 3.1 A high‐throughput chemical screening for the compounds important for in vitro maintaining of hTSCs

First, to understand the role of the chemicals, including CHIR99021 (a WNT agonist), A83‐01 (a TGFβ inhibitor), SB431542 (a TGFβ inhibitor), VPA (an HDAC inhibitor), Y27632 (a ROCK inhibitor) and EGF, supplemented in current culture medium (TSM) for hTSC maintenance,^4^, ^10^ we performed a minus‐one experiment and evaluated the cell viability using Cell Counting Kit‐8 (CCK‐8) assay (Figure S1A). We found that EGF and Y27632 were indispensable for hTSC maintenance (Figure S1A). The removement of CHIR99021, VPA, A83‐01 and SB431542 reduced the hTSC viability, which indicated the vital role of activating WNT and inhibiting TGFβ and HDAC signalling pathways for hTSC maintenance in vitro (Figure S1A).

**FIGURE 1:**
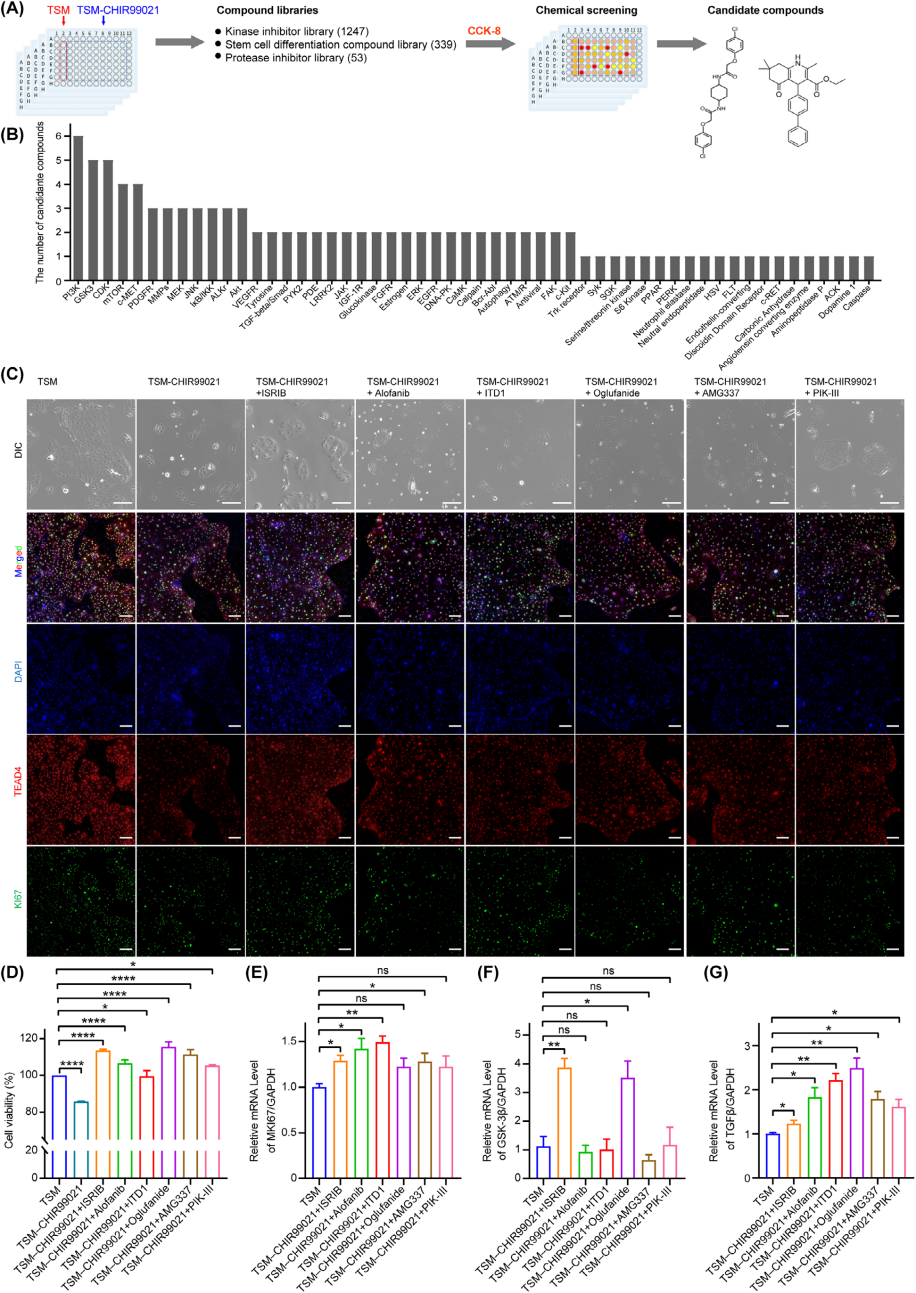
A high‐throughput chemical screening for the compounds important for in vitro maintaining of hTSCs. (A) A schematic diagram of the workflow of CHIR99021‐free screening strategy to identify the candidate chemicals improving hTSC viability. A total of 1639 chemicals was screened and the cell viability was evaluated by CCK‐8 assay. (B) The enrichment of the targets of the candidate chemicals. After the first‐round screening, we obtained 76 candidate chemicals. (C) The representative images of bright field and the immunofluorescence staining for KI67 (a cell proliferative marker protein) and TEAD4 (a hTSC stemness marker protein) in the hTSCs cultured in the indicated mediums. Nuclei were stained by DAPI. Scale bars: 100 μm. (D) The cell viability of the hTSCs cultured in indicated mediums was detected by CCK‐8 assay. Data in this figure are shown as the mean ± SD. *n* = 6. *, *p* < 0.05. ****, *p* < 0.0001. (E–G) Relative expression levels of *KI67/MKI67* (a cell proliferation marker gene), *GSK‐3β* (a WNT signalling pathway‐related gene), *TGFβ* (a TGFβ signalling pathway‐related gene) in the hTSCs cultured in the indicated mediums. Data were shown as the mean ± SD. *n* = 3. *, *p* < 0.05. **, *p* < 0.01. ns, no significance. TSM, hTSC culture medium. TSM‐CHIR99021, remove CHIR99021 in TSM. TSM‐CHIR99021 + ISRIB, remove CHIR99021 and add 0.5 μM ISRIB in TSM. TSM‐CHIR99021 + Alofanib, remove CHIR99021 and add 0.5 μM Alofanib in TSM; TSM‐CHIR99021 + ITD1, remove CHIR99021 and add 0.5 μM ITD1 in TSM. TSM‐CHIR99021 + Oglufanide, remove CHIR99021 and add 0.5 μM Ogluganide in TSM. TSM‐CHIR99021 + AMG337, remove CHIR99021 and add 0.5 μM AMG337 in TSM. TSM‐CHIR99021 + PIK‐III, remove CHIR99021 and add 0.5 μM PIK‐III in TSM. hESC, human embryonic stem cell; hTSC, human trophoblast stem cell.

Then, to overcome the dependence on WNT agonists and TGFβ inhibitors in hTSC medium, we first identified candidate compounds to replace WNT agonist (CHIR99021). By CCK‐8 analysis, we demonstrated a significant decrease in hTSC viability after 4 days of culturing in the TSM without addition of CHIR99021 (Figure S1B). Then, using this CHIR99021‐free culture condition, we performed a high‐throughput screening, which included 1639 compounds consisting of 339 stem cell differentiation compounds, 1247 kinase inhibitors and 53 protease inhibitors, and evaluated the cell viability by CCK‐8 assay after 4 days (Figure 1A). All the chemicals for our first‐round screening were used at a concentration of 1 μM. A total of 76 candidate chemicals were obtained after the first‐round screening, which can improve hTSC viability (Figure S1C). The candidate chemicals targeted multiple signalling pathways known to be important for hTSC maintenance, such as PI3K, GSK‐3β, CDK, mTOR, FGF2, VEGFR, TGFβ and c‐MET, which indicated the reliability of the screening^20^, ^21^, ^22^, ^23^, ^24^, ^25^, ^26^, ^27^ (Figure 1B and Figure S1C).

To further validate the generalisability of the candidate chemicals for hTSC maintenance, we performed a second‐round screening for the 76 candidate chemicals to evaluate their roles in the maintenance of two hTSC lines, including the hTSC line established by our lab and the hTSC line bTS11 gifted by Arima lab. We identified the effects of these candidates on hTSCs at three concentrations, 0.5, 1 and 5 μM. Six chemicals were found to have a strong enhancement for hTSC viability in both hTSC lines (Figure 1C and Figure S1D). Five of these chemicals have been reported to be involved in the induction or maintenance of hTSCs, including Alofanib (a FGFR2 inhibitor), ITD1 (a TGFβ inhibitor), Oglufanide (a VEGF inhibitor), PIK‐III (a VPS34 enzyme inhibitor) and AMG337 (a c‐MET kinase inhibitor) (Figure 1C and Figure S1D). Notably, ISRIB, an inhibitor of integrated stress response,^28^, ^29^, ^30^, ^31^ which showed the most pronounced effect on promoting hTSC viability (Figure 1C,D). Immunofluorescence (IF) staining and quantitative real‐time PCR (qRT‐PCR) for Ki67 showed that ISRIB promoted hTSC proliferation (Figure 1C,E). Furthermore, we found that compared to the hTSCs cultured in TSM, the hTSCs cultured in the TSM in which the WNT agonist was replaced with ISRIB exhibited a higher expression of *GSK‐3β*, an important gene in WNT/β‐Catenin signalling pathway, and an almost unchanged TGFβ expression (Figure 1F,G). Taken together, we found that ISRIB promoted in vitro maintenance of hTSCs and can replace the dependence on WNT agonists in TSM.

### 3.2 ISRIB can maintain the stemness and differentiation capacity of hTSCs

**FIGURE 2:**
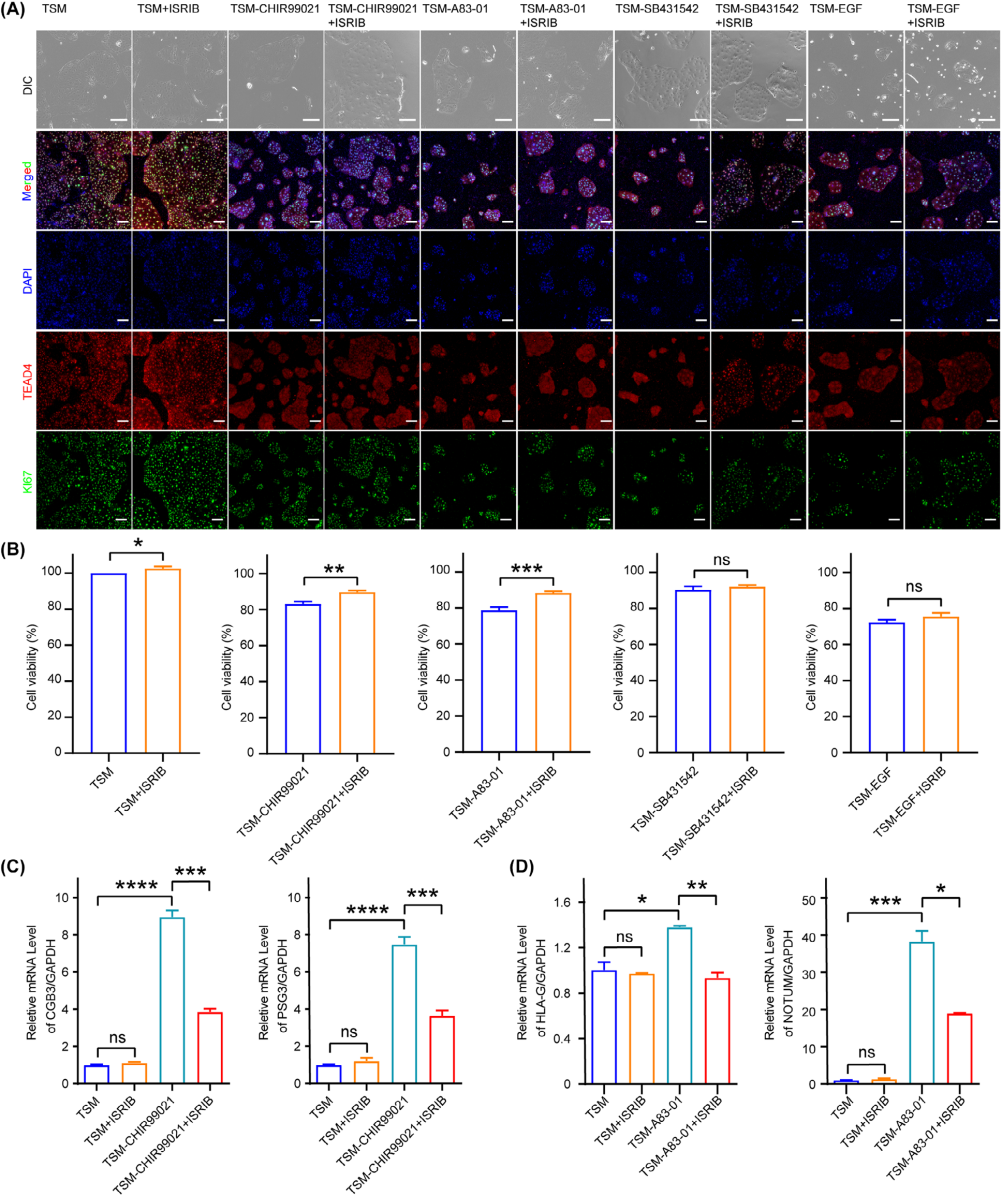
ISRIB can maintain the stemness of hTSCs. (A) The representative images of bright field and the immunofluorescence staining for KI67 (a cell proliferative marker protein) and TEAD4 (a hTSC stemness marker protein) in the hTSCs cultured in the indicated mediums. Nuclei were stained by DAPI. Scale bars: 100 μm. (B) The cell viability of the hTSCs cultured in indicated mediums was detected by CCK‐8 assay. Data were shown as the mean ± SD. *n* = 6. *, *p* < 0.05. **, *p* < 0.01. ***, *p* < 0.001. ns, no significance. (C) Relative expression levels of STB marker genes, *CGB3* and *PSG3*, in the hTSCs cultured in indicated mediums. Data were shown as the mean ± SD. *n* = 3. ***, *p* < 0.001. ****, *p* < 0.0001. ns, no significance. (D) Relative expression levels of EVT marker genes, *HLA‐G* and *NOTUM*, in the hTSCs cultured in the indicated mediums for one passage. Data were shown as the mean ± S. n = 3. *, *p* < 0.05. **, *p* < 0.01. ***, *p* < 0.001. ns, no significance. TSM + ISRIB, add 0.5 μM ISRIB in TSM. TSM‐CHIR99021, remove CHIR99021 in TSM. TSM‐CHIR99021 + ISRIB, remove CHIR99021 and add 0.5 μM ISRIB in TSM. TSM‐A83‐01, remove A83‐01 in TSM. TSM‐A83‐01 + ISRIB, remove A83‐01 and add 0.5 μM ISRIB in TSM. TSM‐SB431542, remove SB431542 in TSM. TSM‐SB431542 + ISRIB, remove SB431542 and add 0.5 μM ISRIB in TSM. TSM‐EGF, remove EGF in TSM. TSM‐EGF + ISRIB, remove EGF and add 0.5 μM ISRIB in TSM. hESC, human embryonic stem cell; hTSC, human trophoblast stem cell.

To further investigate which of the compounds supplemented in TSM can be replaced by ISRIB, we added ISRIB to the minus‐one experiment and evaluated clonal morphology of hTSCs after 4 days of culture. We found that hTSC clones became flattened in the absence of CHIR99021, and the removal of A83‐01 resulted in a significant reduction in the size and number of hTSC clones (Figure 2A and Figure S2A,B). No significant changes were found in the clonal morphology of hTSCs when cultured in the TSM without SB431542, which suggested that SB431542 was not necessary for hTSC maintenance (Figure 2A and Figure S2C). The absence of EGF resulted in almost no complete cell clones (Figure 2A and Figure S2D). By addition of ISRIB to the minus one experiment, we found that ISRIB could significantly rescue the undesirable changes in clonal morphology resulting from the absence of CHIR99021 and A83‐01 (Figure 2A,B). The disruption of cell viability caused by lack of EGF could not be ameliorated by ISRIB (Figure 2B). Furthermore, we plotted the growth curve of hTSCs by successive passages to evaluate the role of CHIR99021, A83‐01, SB431542, EGF and ISRIB on hTSC proliferation. The absence of CHIR99021, A83‐01 or EGF repressed the growth rate of hTSCs. After addition of ISRIB, we found that the depressed proliferation of hTSCs caused by the absence of CHIR99021 or A83‐01 could be rescued, while ISRIB could not rescue the attenuated proliferation of hTSCs caused by the absence of EGF (Figure S2A). Taken together, we demonstrated that ISRIB could replace the use of CHIR99021 and A83‐01 in TSM to maintain hTSC proliferation, and the SB43542 was not required for hTSC maintenance.

Then, we explored whether the hTSC stemness could be maintained after replacing the CHIR99021 and A83‐01 with ISRIB in TSM. First, we detected the effect of the absence of WNT agonist on hTSC stemness, and we found increased expressions of STB marker genes (*CGB3* and *PSG3*)^32^ in the hTSCs cultured in CHIR99021‐free TSM for 6 days, which suggested that the removal of CHIR99021 resulted in a syncytialisation tendency (Figure 2C). We demonstrated that the addition of ISRIB could maintain the stemness of hTSCs after removing CHIR99021 from TSM (Figure 2C). Second, we examined the effect of the absence of TGFβ inhibitor on hTSC stemness, and we found that upregulated expressions of EVT marker genes *HLA‐G* and *NOTUM* ^33^ in the hTSCs cultured in the A83‐01‐free TSM for 6 days, which could also be rescued by the addition of ISRIB. Taken together, the hTSC stemness could be maintained after replacing WNT agonists and TGFβ inhibitors with ISRIB in TSM (Figure 2D).

We further verified the effect of ISRIB on hTSC differentiation potential. We supplemented ISRIB into the STB or EVT culture medium (STBM/EVTM). We found that the mRNA expression levels of *HLA‐G* and *NOTUM* showed no significant change in the EVT differentiated in the EVTM supplemented with ISRIB, suggesting that ISRIB had no effect on EVT differentiation (Figure S3A). We also found that the mRNA expression levels of *CGB3* and *PSG3* decreased in the STB differentiated in the STBM supplemented with ISRIB, suggesting that the syncytialisation of STB was inhibited (Figure S3B). Taken together, ISRIB had no effects in differentiating EVT, but could suppress the form of STB.

**FIGURE 3:**
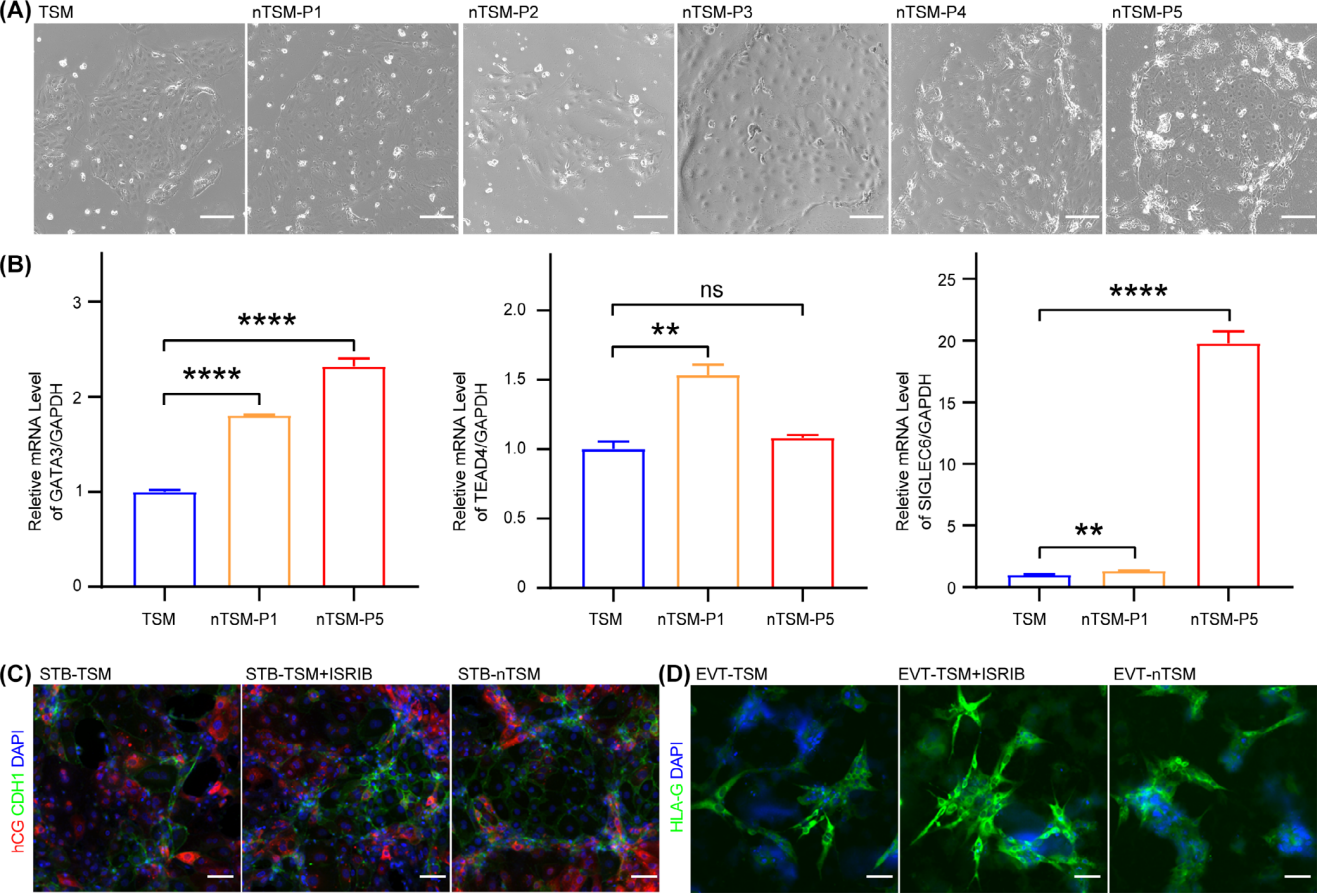
The ISRIB‐dependent nTSM medium can maintain the stemness and differentiation capacity of hTSCs. (A) The representative images of bright field in the hTSCs cultured in TSM or nTSM. P, passage. Scale bars: 100 μm. (B) Relative expression levels of hTSC marker genes, *GATA3*, *TEAD4* and *SIGLEC6*, in the hTSCs cultured in the TSM or nTSM. P, passage. Data were shown as the mean ± SD. *n* = 3. **, *p* < 0.01. ****, *p* < 0.0001. ns, no significance. (C) The representative images of immunofluorescence (IF) staining for hCG and CDH1 protein in the STB derived from the hTSCs cultured in indicated mediums. Nuclei were stained with DAPI. STB‐TSM, STB derived from the hTSCs cultured in TSM. STB‐TSM + ISRIB, STB derived from the hTSCs cultured in the TSM supplemented with 0.5 μM ISRIB, STB‐nTSM, STB derived from the hTSCs cultured in nTSM. Scale bars: 100 μm. (D) The representative images of the IF staining for HLA‐G protein in the EVT derived from the hTSCs cultured in indicated mediums. Nuclei were stained with DAPI. EVT‐TSM, EVT derived from the hTSCs cultured in TSM. EVT‐TSM + ISRIB, EVT derived from the hTSC cultured in the TSM supplemented with 0.5 μM ISRIB. EVT‐nTSM, EVT derived from the hTSCs cultured in nTSM. Scale bars: 100 μm. hESC, human embryonic stem cell; hTSC, human trophoblast stem cell.

Based on the above results, we developed a new culture medium for hTSCs consisting of ISRIB, EGF, VPA and Y27632, namely nTSM. After culturing in nTSM for five passages, we demonstrated that the hTSCs maintained normal clonal growth and morphology (Figure 3A). To identify the cell identity of hTSCs, we detected the expression of trophoblast marker genes, including *GATA3*, *TEAD4* and *SIGLEC6*, which remained highly expressed after passaging in nTSM (Figure 3B). *GATA3* and *TEAD4* are pan CTB markers,^34^, ^35^ and *SIGLEC6* was reported to be expressed in post‐implantation stage.^14^, ^36^ This phenomenon might suggest that hTSCs passaged in the nTSM were more closely resembled post‐implantation trophoblast cells. To evaluate whether the hTSCs cultured in nTSM had normal differentiation capability, we induced the hTSCs cultured in nTSM to differentiate towards STB and EVT, which showed a normal differentiation process and expressed corresponding marker genes (Figure 3C,D). Taken together, we demonstrated that the ISRIB‐dependent nTSM could maintain the stemness and differentiation capacity of hTSCs in the absence of WNT agonists and TGFβ inhibitors for at least five generations.

### 3.3 The hTSCs cultured in nTSM exhibit a similar transcriptional profile to the hTSCs cultured in TSM

**FIGURE 4:**
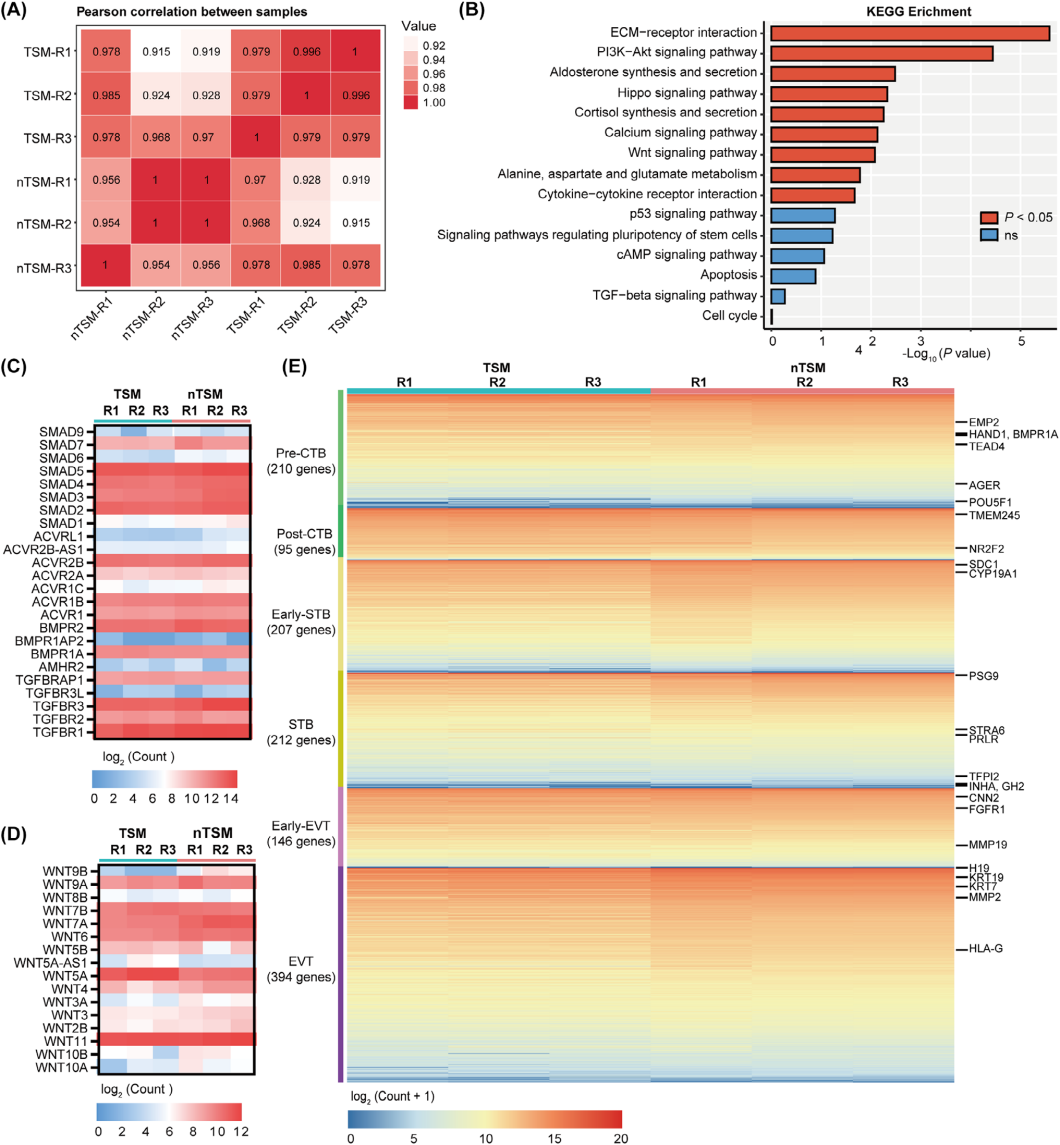
The hTSCs cultured in nTSM exhibit a similar transcriptional profile to the hTSCs cultured in TSM. (A) Correlation analysis of the hTSCs cultured in TSM and nTSM. R, repetition. (B) Kyoto Encyclopedia of Genes and Genomes (KEGG) enrichment of the signalling pathways for differentially expressed genes in the hTSCs cultured in TSM and nTSM. The blue terms represented no significance (ns). The red terms represented *p* < 0.05. (C) Heat map showing the expression (log_2_ count) of TGFβ signalling pathway‐related genes in the hTSCs cultured in TSM and nTSM. R, repetition. (D) Heat map showing the expression (log_2_ count) of WNT signalling pathway‐related genes in the hTSCs cultured in TSM and nTSM. R, repetition. (E) Heat map showing the expression (log_2_(count+1)) of the functional and marker genes of pre‐CTB, post‐CTB, Early‐STB, STB, Early‐EVT and EVT cells in the hTSC cultured in TSM and nTSM. Some indicted genes were shown on the right. hESC, human embryonic stem cell; hTSC, human trophoblast stem cell; R, repetition.

To further characterise the transcriptional similarity between the hTSCs cultured in nTSM and TSM, we performed bulk RNA‐seq on these cells. Correlation analysis showed a high similarity between the hTSCs cultured under these two conditions (Figure 4A). KEGG analysis showed that the hTSCs cultured in nTSM were highly enriched for the signalling pathways important for hTSC maintenance and function, including ECM‐receptor interaction, Hippo signalling pathway, and some metabolic‐related pathways, compared to the hTSCs cultured in TSM (Figure 4B and Figure S4).^37^, ^38^ Although no chemicals targeting WNT and TGFβ signalling pathways were involved in nTSM, KEGG analysis showed that the hTSCs cultured in nTSM exhibited a higher expression level of WNT signalling pathway‐related genes and a comparable expression level of TGFβ signalling pathway‐related genes compared to the hTSCs cultured in TSM (Figure 4B). We also compared the expression of the representative genes belonging to WNT and TGFβ signalling pathways in the hTSCs cultured in nTSM and TSM, which showed almost unchanged (Figure 4C,D). Moreover, the hTSCs cultured in nTSM also showed no significant differences in the signalling pathways regulating hTSC stemness, such as p53 signalling, cAMP signalling, apoptosis, and cell cycle pathways (Figure 4B).

To further confirm the trophoblast cell identity of the hTSCs cultured in nTSM, we compared the expression of the functional and marker genes of trophoblast cells, including pre‐cytotrophoblast (pre‐CTB), post‐CTB, early‐STB, STB, early‐EVT and EVT in the hTSCs cultured in nTSM and TSM (Figure 4E). We found that similar expression levels of the important trophoblast genes in the hTSCs cultured in nTSM and TSM (Figure 4E). Taken together, we demonstrated that the hTSCs cultured in ISRIB‐dependent nTSM had a similar transcriptome profile to those cultured in TSM.

### 3.4 ISRIB can maintain the stemness of hESCs and improve the co‐culture of hESCs and hTSCs

**FIGURE 5:**
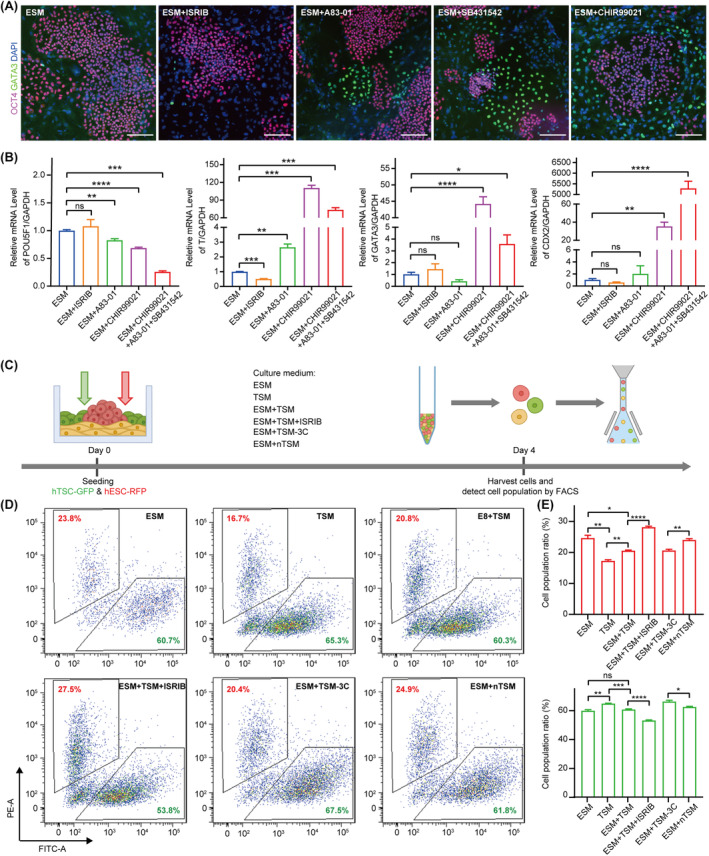
ISRIB can maintain the stemness of hESCs and improve the 2D co‐culture of hESCs and hTSCs. (A) The representative images of the immunofluorescence staining for OCT4 (a pluripotent marker protein) and GATA3 (a trophoblast marker protein) in the hESCs cultured in the ESM supplemented with 0.5 μM ISRIB, 0.5 μM A83‐01, 1 μM SB431542 and 2 μM CHIR99021, respectively, for 6 days. Scale bars: 100 μm. (B) Relative expression levels of *POU5F1*, *T* (a mesodermal lineage marker gene), *GATA3*, and *CDX2* (a trophoblast marker gene) in the hESCs cultured in indicated mediums for 4 days. Data were shown as the mean ± SD. *n* = 3. *, *p* < 0.05. **, *p* < 0.01. ***, *p* < 0.001. ****, *p* < 0.0001. ns, no significance. (C) The schematic diagram of hTSC and hESC 2D co‐culture strategy. hTSC‐GFP, the hTSCs expressing green fluorescent protein. hESC‐RFP, the hESCs expressing red fluorescent protein. (D) The proportion of the hTSC‐GFP and hESC‐RFP cultured in indicated co‐culture mediums was detected by FACS. (E) Bar graphs showing the cell population ratio of the hESC‐RFP (upper panel) and hTSC‐GFP (below panel) cultured in indicated co‐culture mediums. Data were shown as the mean ± SD. *n* = 3. *, *p* < 0.05. **, *p* < 0.01. ***, *p* < 0.001. ****, *p* < 0.0001. ns, no significance. ESM + ISRIB, add 0.5 μM ISRIB in ESM. ESM + A83‐01, add 0.5 μM A83‐01 in ESM. ESM + SB431542, add 1 mM SB431542 in ESM. ESM + CHIR99021, add 2 μM CHIR99021 in ESM. ESM + A83‐01 + SB431542 + CHIR99021, add 0.5 μM A83‐01, 1 μM SB431542 and 2 μM CHIR99021 in ESM. ESM + TSM, 1:1 mixture of ESM and TSM. ESM + TSM + ISRIB, add 0.5 μM ISRIB in 1:1 mixture of ESM and TSM. ESM + TSM‐3C, remove A83‐01, SB431542 and CHIR99021 in 1:1 mixture of ESM and TSM. ESM + nTSM, 1:1 mixture of ESM and nTSM. FACS, flow cytometry; hESC, human embryonic stem cell; hTSC, human trophoblast stem cell.

Since ISRIB could overcome the dependence on WNT agonists and TGFβ inhibitors for hTSC maintenance, we further explored whether ISRIB affected the hESC stemness. We found that the addition of WNT agonist (CHIR99021) or TGFβ inhibitor (SB431542 or A83‐01) induced the expression of extra‐embryonic lineage marker gene *GATA3* in hESCs by IF, which was not observed in the hESCs cultured in the medium supplemented with ISRIB (Figure 5A). Moreover, by qRT‐PCR analysis, we found that the addition of WNT agonist or TGFβ inhibitor reduced the expression of pluripotency gene *POU5F1* (*OCT4*), while upregulated the expression of mesodermal lineage marker gene *BRACHURY* (*T*) in hESCs, which suggested a disrupted hESC stemness (Figure 5B). We confirmed that the addition of ISRIB in hESC medium (ESM) had no effect on the expression of pluripotency, extra‐embryonic lineage and mesodermal lineage marker genes (Figure 5B). Taken together, these results showed that the addition of ISRIB could maintain hESC stemness.

Then, we explored whether ISRIB could promote the co‐culture of hESCs and hTSCs. We established a 2D co‐culture reporter system using hTSC expressing GFP (hTSC‐GFP) and hESC expressing RFP (hESC‐RFP). After a 4‐day co‐culture of hESC‐RFP and hTSC‐GFP within indicated culture conditions, the cell proportion of hESCs and hTSCs was evaluated by flow cytometry analysis (Figure 5C). By using this system, we examined the development of hESCs and hTSCs under six culture conditions, including ESM, TSM, ESM + TSM, ESM + TSM + ISRIB, ESM + TSM‐3C and ESM + nTSM (Figure 5C–E). First, we analysed the proliferation of hESCs in the indicated co‐culture mediums (Figure 5D,E). We found that the addition of TSM (TSM and ESM + TSM) significantly inhibited hESC growth (Figure 5D,E). After removing WNT agonists and TGFβ inhibitors from TSM (ESM + TSM‐3C), the inhibition of hESC proliferation by TSM was alleviated (Figure 5D,E). The addition of ISRIB promoted the growth of hESCs (ESM + nTSM) (Figure 5D,E). Then, we analysed the proliferation of the hTSCs cultured in indicated co‐culture mediums (Figure 5D,E). We found that the addition of ESM slightly inhibited hTSC proliferation, suggesting that hTSCs were more tolerant to culture conditions than hESCs (Figure 5D,E). Taken together, among the six indicated media, the ESM + nTSM maintained the most balanced growth of hESCs and hTSCs in the 2D co‐culture system.

### 3.5 ISRIB maintains the cell fate of hESCs and hTSCs in the 2D co‐culture system

**FIGURE 6:**
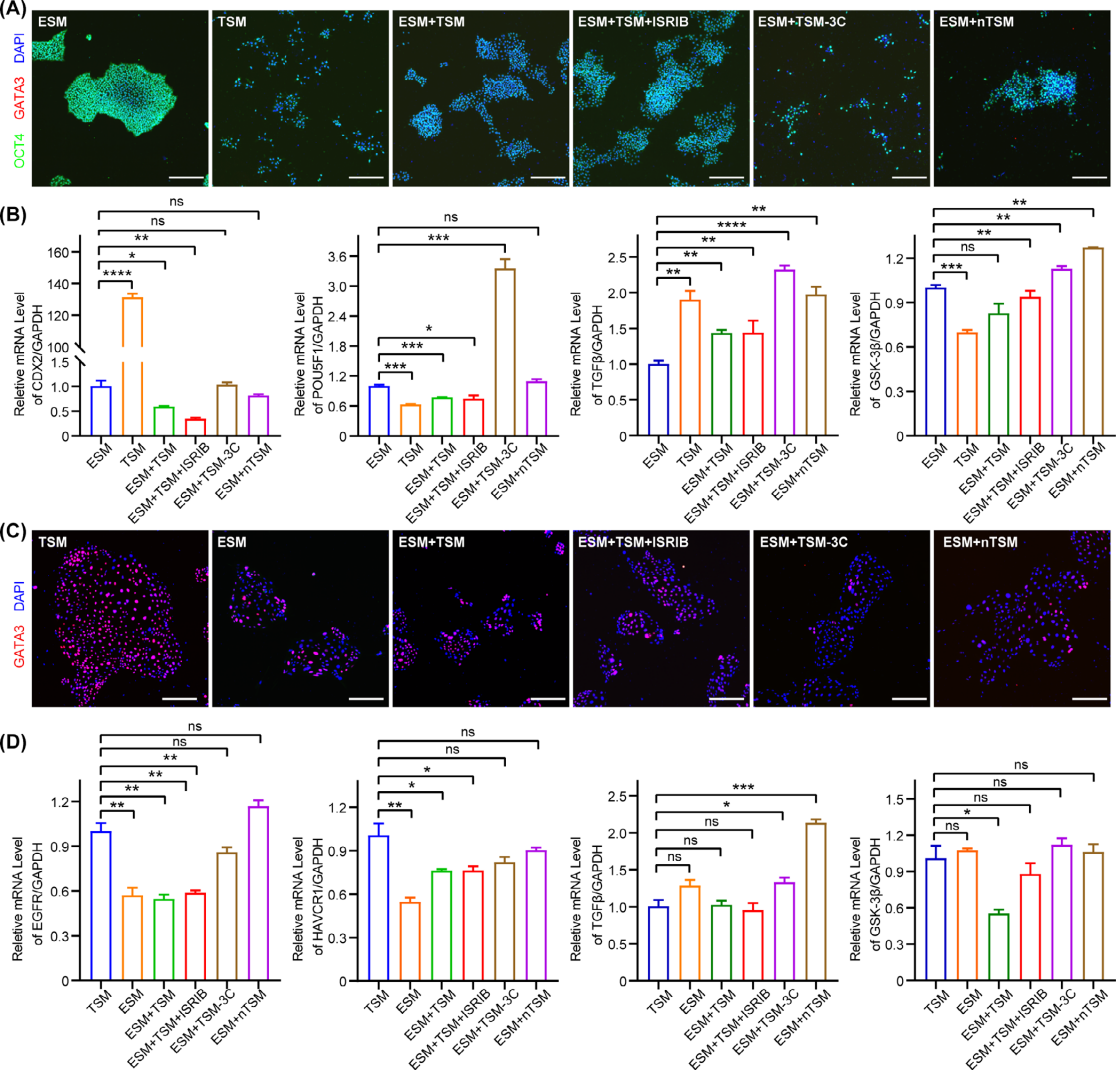
ISRIB maintains the cell fate of hESCs and hTSCs in the 2D co‐cultured system. (A) The representative images of the immunofluorescence (IF) staining for OCT4 and GATA3 in the hESCs cultured in indicated mediums for 4 days. Nuclei were stained with DAPI. Scale bars: 100 μm. (B) Relative expression levels of *CDX2*, *POU5F1*, *TGFβ* and *GSK‐3β* in the hESCs cultured in indicated mediums. Data were shown as the mean ± SD. *n* = 3. *, *p* < 0.05. **, *p* < 0.01. ***, *p* < 0.001. ****, *p* < 0.0001. ns, no significance. (C) The representative images of the IF staining for GATA3 in the hTSCs cultured in indicated mediums for 4 days. Nuclei were stained with DAPI. Scale bars: 100 μm. (D) Relative expression levels of *EGFR*, *HAVCR1*, *TGFβ* and *GSK‐3β* in the hTSCs cultured in indicated mediums. Data were shown as the mean ± SD. *n* = 3. *, *p* < 0.05. **, *p* < 0.01. ***, *p* < 0.001. ****, *p* < 0.0001. ns, no significance. ESM + TSM, 1:1 mixture of ESM and TSM. ESM + TSM + ISRIB, add 0.5 μM ISRIB in 1:1 mixture of ESM and TSM. ESM + TSM‐3C, remove A83‐01, SB431542 and CHIR99021 in 1:1 mixture of ESM and TSM. ESM + nTSM, 1:1 mixture of ESM and nTSM. hESC, human embryonic stem cell; hTSC, human trophoblast stem cell.

To further verify the maintenance of hESCs and hTSCs in the co‐culture system, we cultured hESCs and hTSCs in indicated co‐culture mediums, respectively, and characterised the cell fate of hESCs and hTSCs. First, we observed the clonal morphology of the hESCs cultured in the indicated co‐culture medium, and complete hESC clones were barely observed when cultured in TSM (Figure 6A), which could not be rescued by removing WNT agonists and TGFβ inhibitors (ESM + TSM‐3C) (Figure 6A). Nevertheless, we found that the clonal morphology was significantly improved by the addition of ISRIB (ESM + TSM + ISRIB and ESM + nTSM) (Figure 6A). Then, we detected the expression of pluripotency gene *POU5F1* in hESCs by IF and qRT‐PCR, which showed a comparable expression level in the hESCs cultured in ESM + nTSM as that in the hESCs cultured in ESM, suggesting the maintenance of hESC stemness by ESM + nTSM (Figure 6B). The addition of nTSM also did not induce the expression of extra‐embryonic genes *GATA3* and *CDX2* in hESCs (Figure 6B). We found that the hESCs cultured in ESM + TSM‐3C showed a lower cell population ratio than those cultured in ESM + TSM. However, when comparing the expression of maker genes, the reduced expression of OCT4 suggests that hESCs cultured in ESM + TSM had a tendency to differentiate. In comparison, the expression level of OCT4 was elevated when hESCs were cultured in ESM + TSM‐3C (Figure 6A,B). Moreover, we detected the expression level of TGFβ and WNT signalling pathway‐related gene in the hESCs cultured in ESM + nTSM, and found that the hESCs maintained highly expression of *TGFβ* and almost unchanged expression of WNT signalling gene *GSK‐3β* compared to those in the hESCs cultured in ESM (Figure 6B). Taken together, we demonstrated that ESM + nTSM maintained the hESC stemness and the important signalling pathways required for hESC maintenance.

Next, we examined the cell fate of the hTSCs cultured in indicated co‐culture mediums. We found that the addition of ESM and removal of WNT agonists and TGFβ inhibitors (ESM and ESM + TSM‐3C) resulted in smaller hTSC clones compared to the hTSCs cultured in TSM, while the hTSCs cultured in ESM + nTSM remained normal clonal morphology and size (Figure 6C). Besides, the hTSCs cultured in ESM + nTSM maintained normal clonal growth. Moreover, we demonstrated that the hTSCs cultured in ESM + nTSM remained stable expression of important marker genes, such as *EGFR*, *HAVCR1* and *GSK‐3β* (Figure 6D). However, we found a slightly increased expression of *TGFβ* in the hTSCs cultured in ESM + nTSM, which suggested that although ESM + nTSM could maintain hTSC clonal morphology, it was not appropriate for long‐term in vitro culture of hTSCs (Figure 6D). Taken together, ISRIB‐dependent ESM + nTSM maintained the cell fate and proliferation of both hESCs and hTSCs in the co‐culture system.

Furthermore, we validated the potential mechanism by which this ISRIB‐dependent system balances hESC and hTSC cultures. ISRIB counteracts toxic chronic ISR activity, without disturbing the cytoprotective effects of a strong acute ISR. It has a promising therapeutic potential in vivo without overt side effects.^28^ We found that the ISR marker *ATF4* was activated in hTSCs cultured in TSM without WNT agonist and TGFβ inhibitors (Figure S4). When supplemented with ISRIB or a selective catalytic ISR inhibitor, GSK2656157,^39^ the expression of *ATF4* was significantly reduced, indicating that ISRIB could redeem the ISR due to WNT deficiency and TGFβ activation, thereby promoting hTSC maintenance (Figure S4). We also found that the expression of *CHOP*, a marker gene downstream of the ISR, was significantly upregulated after the addition of WNT agonists and TGFβ inhibitors to the ESM (Figure S5). Overexpression of *CHOP* has been reported to lead to cell cycle arrest and/or apoptosis.^40^, ^41^ The addition of ISRIB can undo the *CHOP* activation induced by WNT agonist. Furthermore, simultaneous activation of WNT and inhibition of TGFβ leads to severe cellular stress in hESCs, and supplementation with low‐dose ISRIB partially attenuates *CHOP* hyperactivation (Figure S5). Taken together, interfering with the normal expression of WNT and TGFβ in hTSCs and hESCs induces the cellular stress. ISRIB could rescue this process thereby promoting stem cell maintenance.

### 3.6 ISRIB facilitates the organisation of hESC and hTSC aggregates in 3D conditions

**FIGURE 7:**
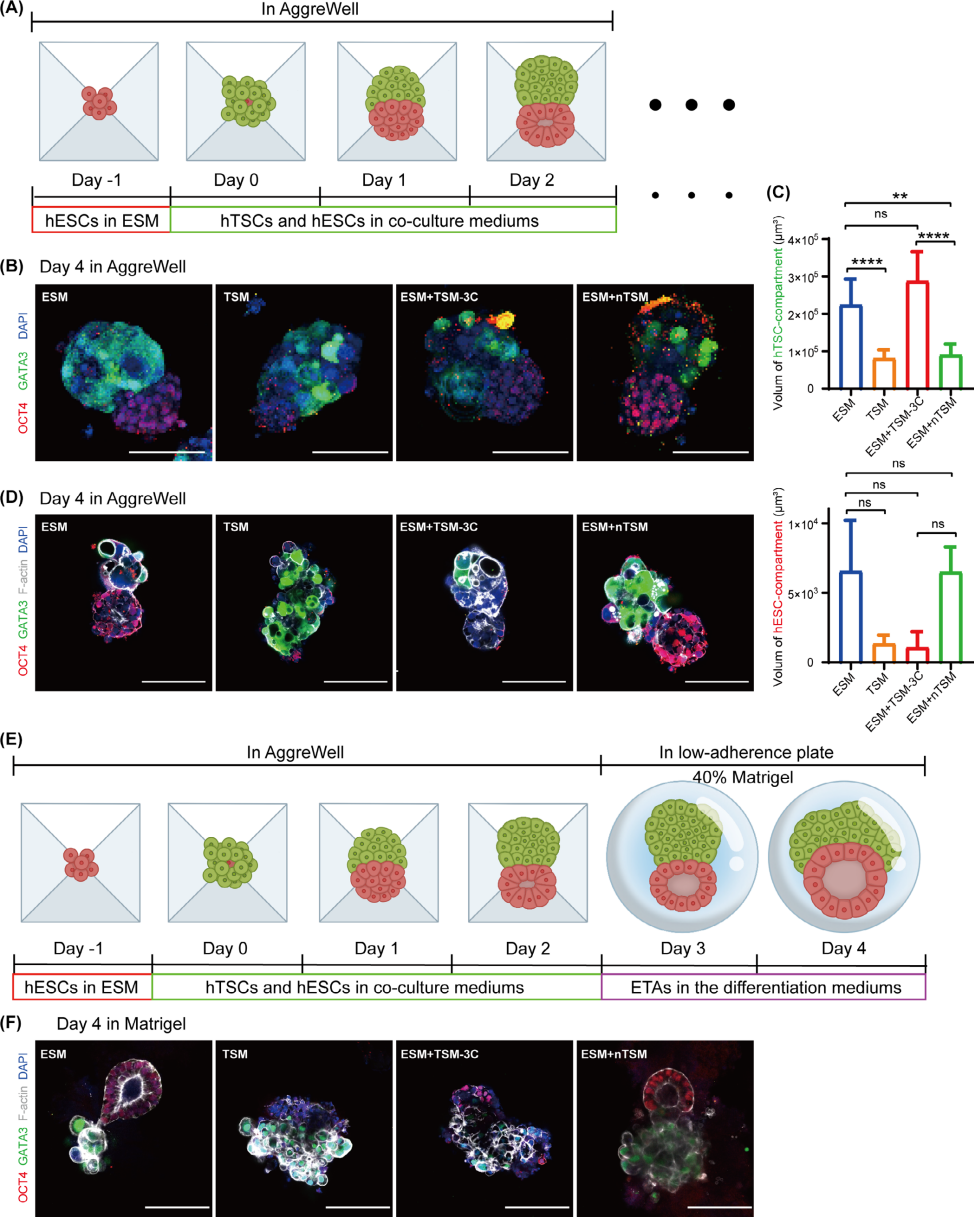
ISRIB facilitates the organisation of hESC and hTSC aggregates in 3D conditions. (A) The schematic diagram of constructing 3D hESCs and hTSCs aggregates (ETAs). The ETAs were cultured in indicated co‐culture mediums, respectively. (B) The representative images of the immunofluorescence (IF) staining for OCT4 (hESC‐compartment) and GATA3 (hTSC‐compartment) in the ETAs cultured in indicated co‐culture mediums. Nuclei were stained with DAPI. Scale bars: 100 μm. (C) The analysis of the volume of hTSC‐compartments (upper panel) and hESC‐compartments (below panel) in the ETAs cultured in indicated mediums. Data were shown as the mean ± SD. *n* ≥ 3. *, *p* < 0.05. **, *p* < 0.01. ***, *p* < 0.001. ****, *p* < 0.0001. ns, no significance. (D) The representative images of the IF staining for F‐actin (a lumenogenesis marker protein), OCT4 and GATA3 in the ETAs cultured in indicated co‐culture mediums. Nuclei were stained with DAPI. Scale bars: 100 μm. (E) The schematic diagram of the strategy supporting continued development of ETAs. From Day −1 to Day 2, the culture strategy was consistent with that in (A). Then, the ETAs were transferred into 40% Matrigel and cultured in differentiation mediums for further development. On Day 3, the ETAs were cultured in APEL2 medium supplemented with 3 μM CHIR99021, 50 ng/mL EGF and 40% Matrigel. On Day 4, the medium was replaced with APEL2 supplemented with 3 μM CHIR99021, 5 ng/mL FGF2 and 50 ng/mL EGF. (F) The representative images of the IF staining for F‐actin, OCT4 and GATA3 in the ETAs at Day 4. Scale bars: 100 μm. The indicated co‐culture mediums included ESM, TSM, ESM + TSM‐3C and ESM + nTSM. ESM + TSM‐3C, remove A83‐01, SB431542 and CHIR99021 in 1:1 mixture of ESM and TSM. ESM + nTSM, 1:1 mixture of ESM and nTSM. hESC, human embryonic stem cell; hTSC, human trophoblast stem cell.

Using our previously established 3D co‐culture system (hESCs and hTSCs aggregates, ETAs),^18^ we further explored whether ESM + nTSM can facilitate the spatial organisation of hESCs and hTSCs. We compared the developmental efficiency of ETAs in indicated co‐culture mediums, including ESM, TSM, ESM + TSM‐3C and ESM + nTSM. First, we examined the effect of the indicated mediums on the assembly efficiency of hESCs and hTSCs. The hESCs were seeded to AgreeWell™ 400 with 14,400 cells per well, and hTSCs were seeded 24 h later with 80,000 cells per well, at which time was defined as Day 0 (Figure 7A). The ETAs were cultured in indicated mediums and changed half daily. We performed IF for OCT4 and GATA3 at Day 4, and analysed the development of hESC‐ and hTSC‐compartments (Figure 7B‐D). We found that the addition of TSM significantly reduced the volume of the hESC‐compartments in ETAs compared to the ETAs cultured in ESM (Figure 7B,C). When the ETAs were cultured in the mediums containing ESM (ESM and ESM + TSM‐3C), the transcription factor GATA3 showed a simultaneous localisation in the nucleus and cytoplasm of the hTSCs in ETAs (Figure 7B). In the hTSCs of the ETAs cultured in ESM + nTSM, GATA3 protein was localised in the nucleus only (Figure 7B). We found that the volume of the hTSC‐compartment of the ETAs cultured in the medium containing ESM (ESM and ESM + TSM‐3C) was larger than that cultured in TSM (Figure 7C). However, IF for F‐actin indicated a number of vacuoles in the hTSC‐compartments of ETAs, suggesting a reduced cell viability of hTSCs, while the hTSC‐compartments developed normally in the ETAs cultured in ESM + nTSM (Figure 7D and Figure S6A). Taken together, we demonstrated that ISRIB‐dependent ESM + nTSM promoted the organisation and maintenance of both hESC‐ and hTSC‐compartments in ETAs.

The epiblast undergoes lumenogenesis to form primary‐amniotic cavity after implantation. To explore whether ESM + nTSM can support the lumenogenesis of hESC‐compartments in ETAs, we transferred the ETAs, which had been cultured in indicated co‐culture mediums for 3 days, to 40% Matrigel and were continued culturing for 2 days in differentiation mediums (Figure 7E). We found that the ETAs cultured in ESM or TSM for 3 days could only support the development of hESC‐ or hTSC‐compartment in Matrigel, respectively (Figure 7F and Figure S6B). The ETAs precultured in ESM + TSM‐3C were able to support the development of both hESC‐ and hTSC‐compartments, while the lumenogenesis in the hESC‐compartments was limited (Figure 7F). By comparison with the above culture mediums, we demonstrated that the ETAs precultured in the ISRIB‐dependent ESM + nTSM exhibited optimal assembly of hESC‐ and hTSC‐compartments and lumenogenesis in hESC‐compartments (Figure 7F and Figure S6B). Taken together, ESM + nTSM facilitated the organisation and development of ETAs.

## 4 DISCUSSION

Establishment of human embryoids by assembling the hESCs and hTSCs, which represent embryonic and extra‐embryonic lineage progenitor cells, respectively, remains challenging due to the lack of appropriate in vitro co‐culture system. The main limitation for co‐culturing hESCs and hTSCs is that the maintenance of hESCs and hTSCs depends on the opposite regulation of some signalling pathways, especially WNT and TGFβ signalling pathways. Here, by performing a high‐throughput chemical screening, we provided a systematic understanding of the signalling pathways for hTSC maintenance in vitro. Notably, we first demonstrated that ISRIB, an inhibitor of integrated stress response, could maintain the stemness and differentiation capacity of hTSCs. Thus, we developed an ISRIB‐dependent medium, nTSM, for hTSC maintenance. Besides, we demonstrated that ISRIB could also maintain hESC stemness. Under co‐culture conditions, the addition of ISRIB redeemed the cellular stress response due to the opposite demand for WNT and TGFβ signalling by hTSCs and hESCs, inhibited cell differentiation and death, and promoted stemness maintenance. Then, by using our previously described ETA system, we developed a 3D co‐culture medium, ESM + nTSM, which could improve the organisation and development of both hESC‐ and hTSC‐compartments under 3D conditions.

Chen et al. have developed a four‐factor cocktail CEPT including ISRIB could promote the viability of hESCs.^42^ Our results also demonstrated that the addition of ISRIB could not only preserve the stemness of both hESCs and hTSCs, but also maintain the expression levels of WNT and TGFβ signalling pathway‐related genes in the absence of chemicals targeting WNT and TGFβ signalling pathways. These results demonstrated that ISRIB played important roles in cell viability and cell fate in both embryonic and extra‐embryonic lineage cells, which suggested that ISRIB could be used as an important candidate compound for developing embryoids using stem cells. The ISRIB‐dependent co‐culture system will advance our understanding of the interactions between embryonic and extra‐embryonic tissues during human early embryonic development.

## AUTHOR CONTRIBUTIONS

L.Y., H.W. and H.M.W. conceived and supervised the project. S.W.X., D.N.Y. and H.W. performed cell culture of hTSCs and hESCs. S.W.X. and H.W. performed chemical screening with the help of Y.W., Y.R. and S.C. S.W.X. performed immunostaining and qRT‐PCR. D.N.Y., H.W. and S.W.X. analysed transcriptome data. S.W.X. and B.J.H. completed the pattern diagrams. S.W.X., D.N.Y. and H.W. completed the figures. S.W.X., H.W. and D.N.Y. wrote the manuscript. All authors contributed to the manuscript revision, and read and approved the submitted version.

## CONFLICT OF INTEREST STATEMENT

The authors declare no conflicts of interest.

## Supporting information


**Figure S1.** Signal enrichment of the target genes of the candidate compounds.
**Figure S2.** ISRIB can improve the proliferation of hTSCs in the absence of WNT activation and TGFβ inhibition.
**Figure S3.** Effects of ISRIB on EVT and STB differentiation.
**Figure S4.** ISRIB can redeem cellular stress induced by WNT agonist and TGFβ inhibitor deficiency in hTSCs.
**Figure S5.** ISRIB can redeem cellular stress induced by WNT activation and TGFβ inhibition in hESCs.
**Figure S6.** ISRIB facilitates the organisation of hESC and hTSC aggregates in 3D conditions.

**Table S1.** Quantitative Real‐Time PCR primer pairs related to this article.

**Table S2.** Marker gene list of trophoblast cells, related to Figure 4.

## Data Availability

The RNA‐seq data in this paper are available at the NCBI Gene Expression Omnibus (GEO) with the accession GSE221409. The data were also deposited to the National Genomics Data Center of China under accession number (HRA004046). The original contribution data presented in the study are included in the article/Supporting Information; further inquiries can be directed to contact with the corresponding author.
