# Supplementary material for: ISRIB facilitates the co‐culture of human trophoblast stem cells and embryonic stem cells
Source: Cell Prolif. 2024 Jan 12;57(6):e13599. doi: 10.1111/cpr.13599 (PMC11150133; doi:10.1111/cpr.13599)
Supplement: Supplementary file 1 — Figure S1. Signal enrichment of the target genes of the candidate compounds. Figure S2. ISRIB can improve the proliferation of hTSCs in the absence of WNT activation and TGFβ inhibition. Figure S3. Effects of ISRIB on EVT and STB differentiation. Figure S4. ISRIB can redeem cellular stress induced by WNT agonist and TGFβ inhibitor deficiency in hTSCs. Figure S5. ISRIB can redeem cellular stress induced by WNT activation and TGFβ inhibition in hESCs. Figure S6. ISRIB facilitates the organisation of hESC and hTSC aggregates in 3D conditions. [file CPR-57-e13599-s003.docx]

**Supplementary Figure 1**


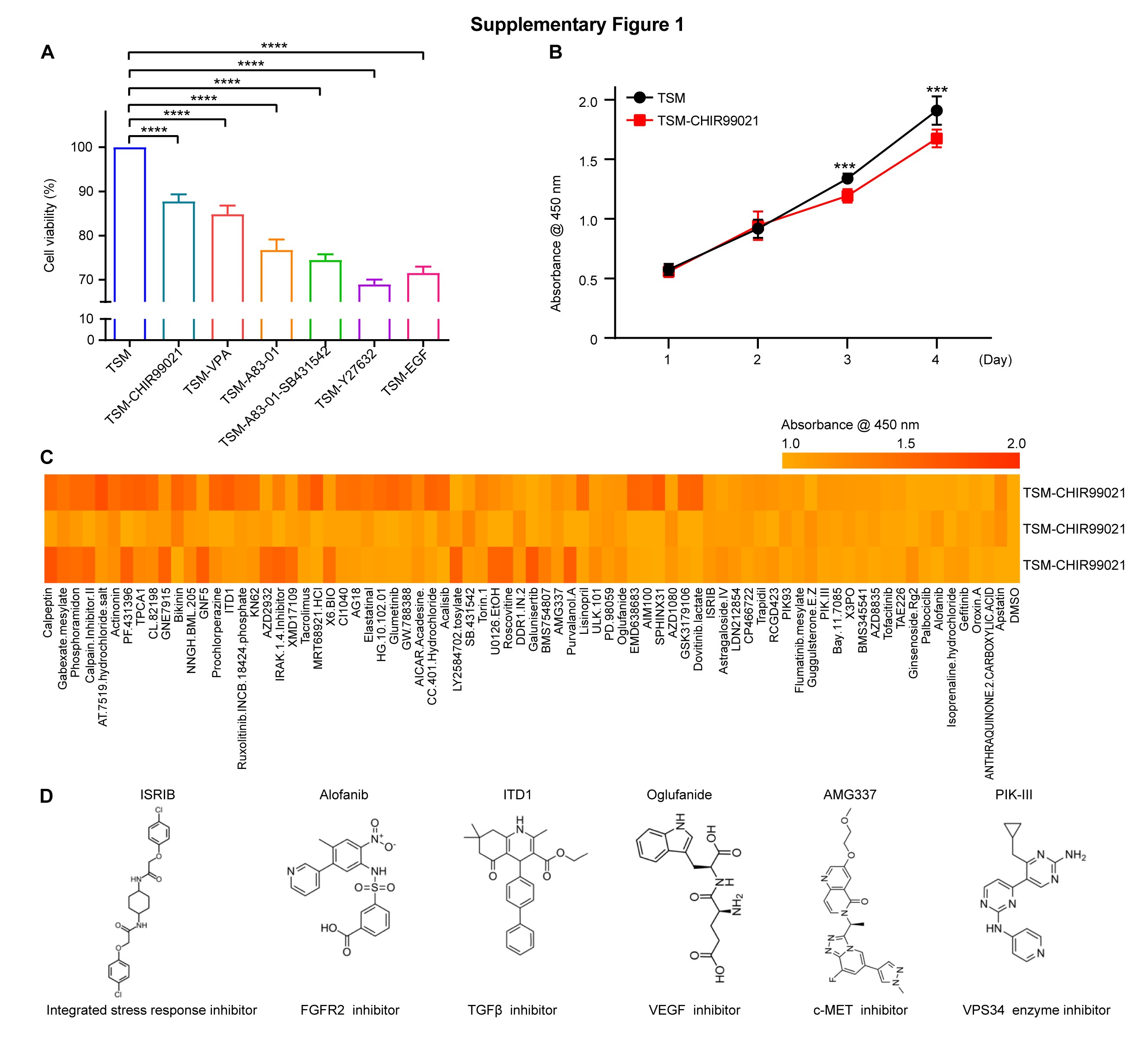


**Figure S1. Signal enrichment of the target genes of the candidate compounds. Related to Figure 1.**

(A) The cell viability of the hTSCs cultured in indicated mediums, which was detect by CCK-8 assay. Data in this figure are shown as the mean ± s.d. n=6. ****, P < 0.0001.

(B) The cell viability of the hTSCs cultured in the TSM-CHIR99021, which was detect by CCK-8 assay. Data were shown as the mean ± s.d. n=6. ***, P < 0.001.

(C) The Heatmap showing the cell viability of the hTSCs cultured in the TSM-CHIR99021 supplemented with 76 candidate compounds.

(D) The structural formula of the candidate compounds after second-round screening. The targeted signal pathway was written below.

TSM-CHIR99021, remove CHIR99021 in TSM. TSM-VPA, remove VPA in TSM. TSM-A83-01, remove A83-01 in TSM. TSM-A83-01-SB431542, remove A83-01 and SB431542 in TSM. TSM-Y27632, remove Y27632 in TSM. TSM-EGF, remove EGF in TSM.

**Supplementary Figure 2**


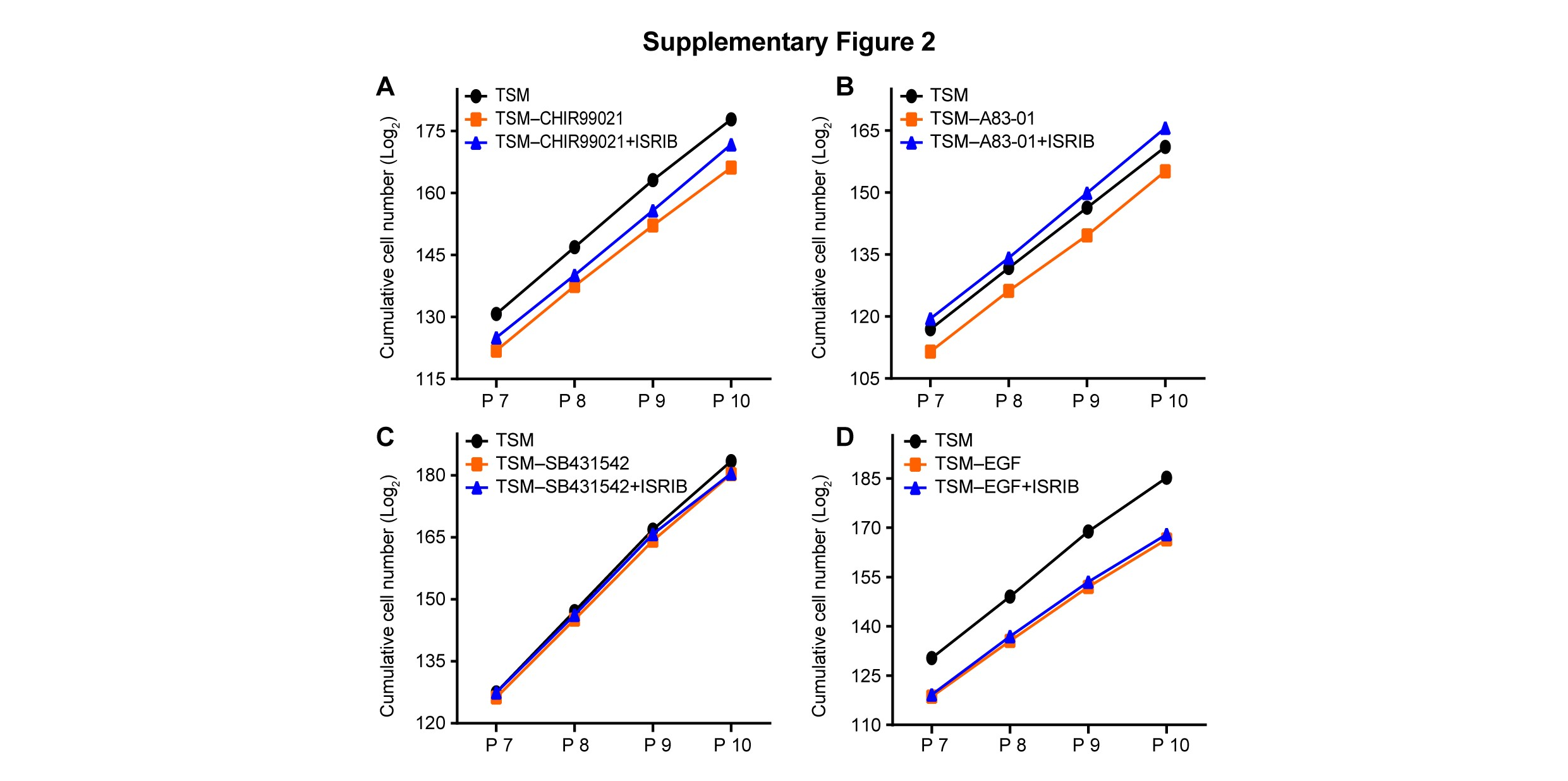


**Figure S2. ISRIB can improve the proliferation of hTSCs in the absence of WNT activation and TGFβ inhibition.** **Related to** **Figure 2.**

(A-D) Growth curve of the hTSCs cultured in indicated mediums. P, passage. TSM+ISRIB, add 0.5 μM ISRIB in TSM. TSM-CHIR99021, remove CHIR99021 in TSM. TSM-CHIR99021+ISRIB, remove CHIR99021 and add 0.5 μM ISRIB in TSM. TSM-A83-01, remove A83-01 in TSM. TSM-A83-01+ISRIB, remove A83-01 and add 0.5 μM ISRIB in TSM. TSM-SB431542: remove SB431542 in TSM. TSM-SB431542+ISRIB, remove SB431542 and add 0.5 μM ISRIB in TSM. TSM-EGF, remove EGF in TSM. TSM-EGF+ISRIB, remove EGF and add 0.5 μM ISRIB in TSM.

**Supplementary Figure 3**


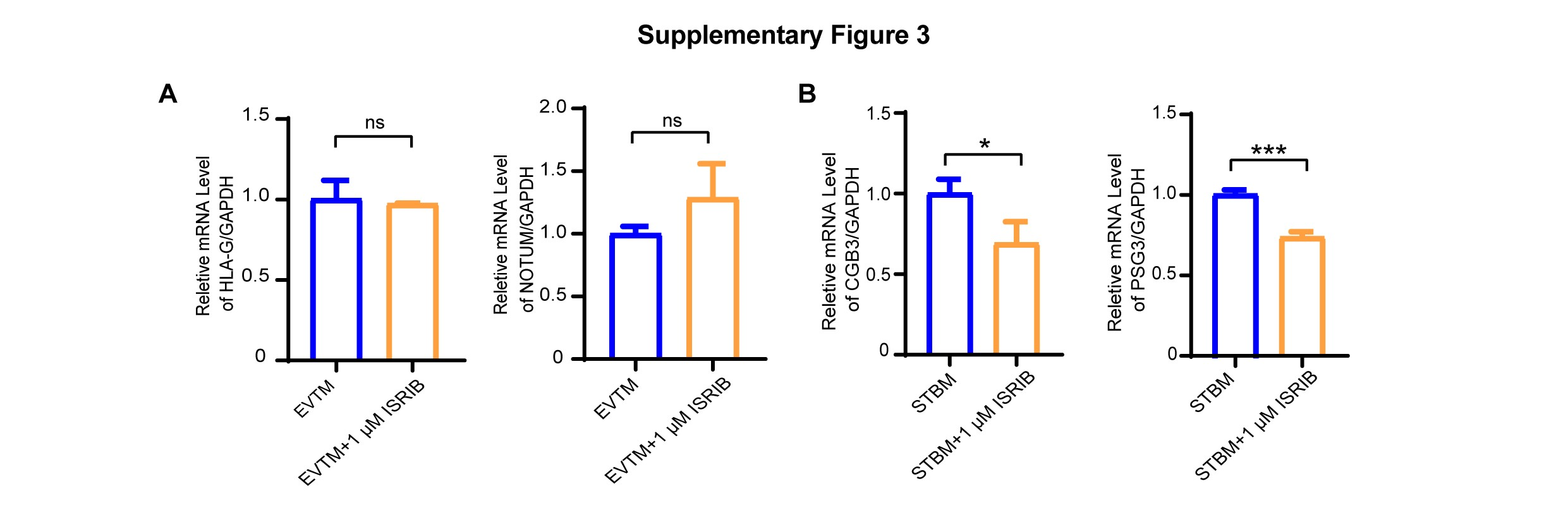


**Figure S3. Effects of ISRIB on EVT and STB differentiation.**

(A) Relative expression levels of *HLA-G* and *NOTUM* in the hTSC derived EVTs cultured in indicated differentiation mediums. Data were shown as the mean ± s.d. n=3. ns, no significance.

(B) Relative expression levels of *CGB3* and *PSG3* in the hTSC derived STB cultured in indicated differentiation mediums. Data were shown as the mean ± s.d. n=3. *, P < 0.05. ***, P < 0.001.

EVTM, EVT differentiation medium. EVTM+1 μM ISRIB, add 1 μM ISRIB in EVTM. STBM, STB differentiation medium. STBM+1 μM ISRIB, add 1 μM ISRIB in STBM.

**Supplementary Figure 4**


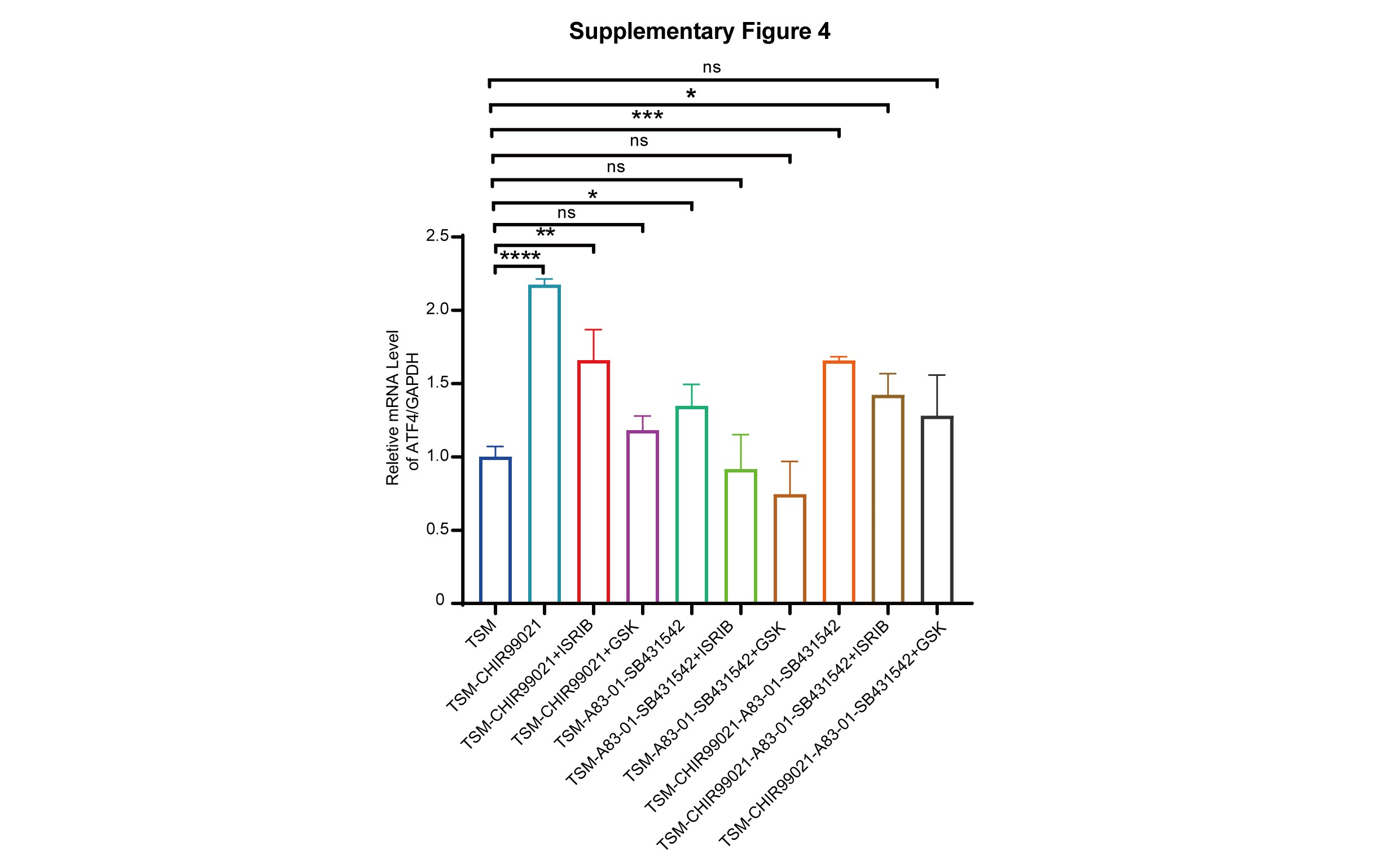


**Figure S4. ISRIB can redeem cellular stress induced by WNT agonist and TGFβ inhibitor deficiency in hTSCs.**

Relative mRNA expression level of *ATF4* in the hTSCs cultured in the indicated mediums for one passage. Data were shown as the mean ± s.d. n=3. *, P < 0.05. **, P < 0.01. ***, P < 0.001. ****, P < 0.0001. ns, no significance. TSM-CHIR99021, remove CHIR99021 in TSM. TSM-CHIR99021+ISRIB, remove CHIR99021 and add 0.5 μM ISRIB in TSM. TSM-CHIR99021+GSK, remove CHIR99021 and add 0.5 μM GSK2656157 in TSM. TSM-A83-01-SB431542, remove A83-01 and SB431542 in TSM. TSM-A83-01-SB431542+ISRIB, remove A83-01 and SB431542, and add 0.5 μM ISRIB in TSM. TSM-A83-01-SB431542+GSK, remove A83-01 and SB431542, and add 0.5 μM GSK2656157 in TSM. TSM-CHIR99021-A83-01-SB431542, remove CHIR99021A, 83-01 and SB431542 in TSM. TSM-CHIR99021-A83-01-SB431542+ISRIB, remove CHIR99021A, A83-01 and SB431542, and add 0.5 μM ISRIB in TSM. TSM-CHIR99021-A83-01-SB431542+GSK, remove CHIR99021A, A83-01 and SB431542, and add 0.5 μM GSK2656157 in TSM.

**Supplementary Figure 5**


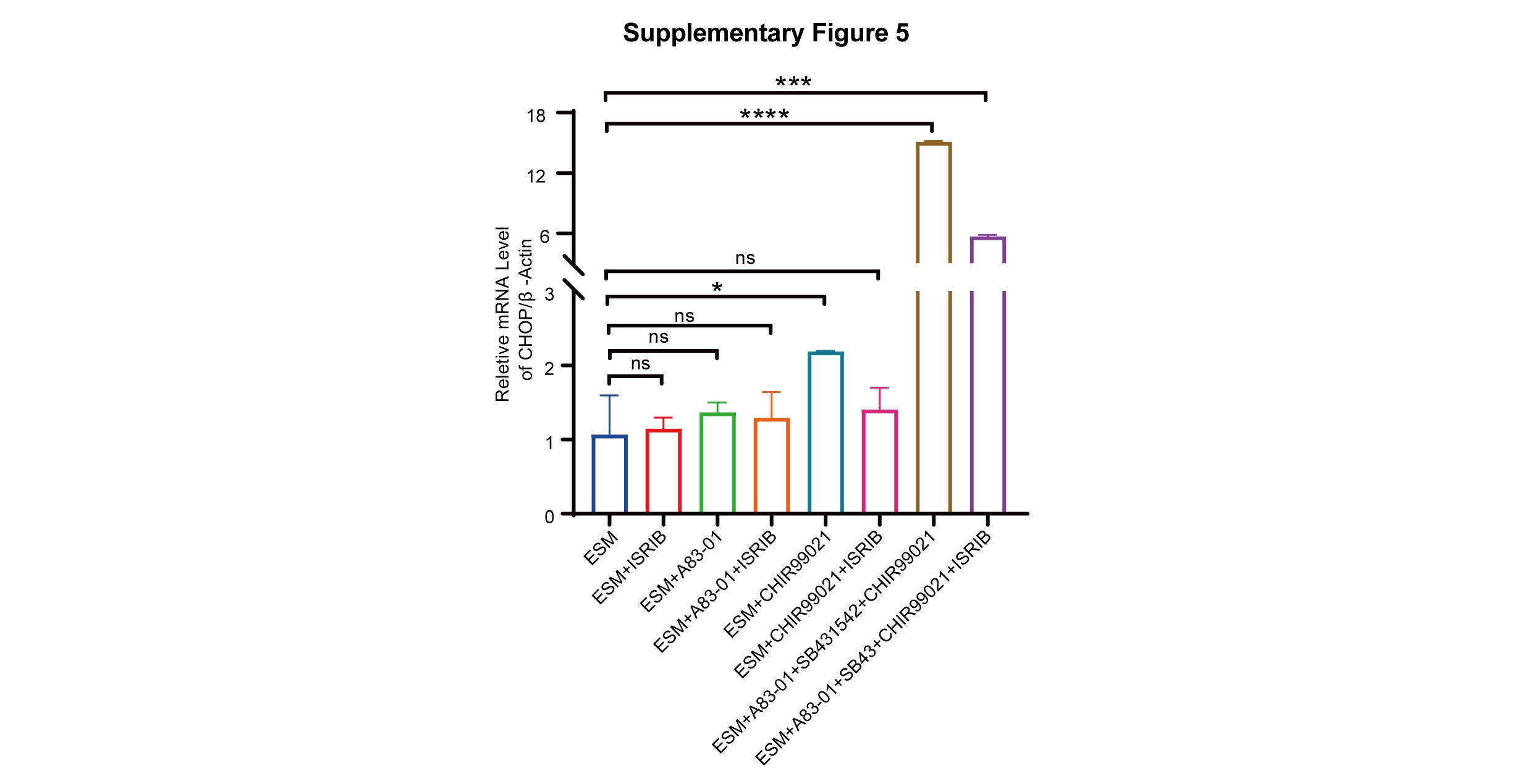


**Figure S5. ISRIB can redeem cellular stress induced by WNT activation and TGFβ inhibition in hESCs.**

Relative mRNA expression level of *CHOP* in the hESCs cultured in the indicated mediums for one passage. Data were shown as the mean ± s.d. n=3. *, P < 0.05. ***, P < 0.001. ****, P < 0.0001. ns, no significance. ESM+ISRIB, add 0.5 μM ISRIB in ESM. ESM+A83-01, add A83-01 in ESM. ESM+A83-01+ISRIB, add A83-01 and 0.5 μM ISRIB in ESM. ESM+CHIR99021, add CHIR99021 in ESM. ESM+CHIR99021+ISRIB, add CHIR99021 and 0.5 μM ISRIB in ESM. ESM+A83-01+SB431542+CHIR99021, add A83-01, SB431542, and CHIR99021 in ESM. ESM+A83-01+SB43+CHIR99021+ISRIB, add A83-01, SB431542, CHIR99021, and 0.5 μM ISRIB in ESM.

**Supplementary Figure 6**


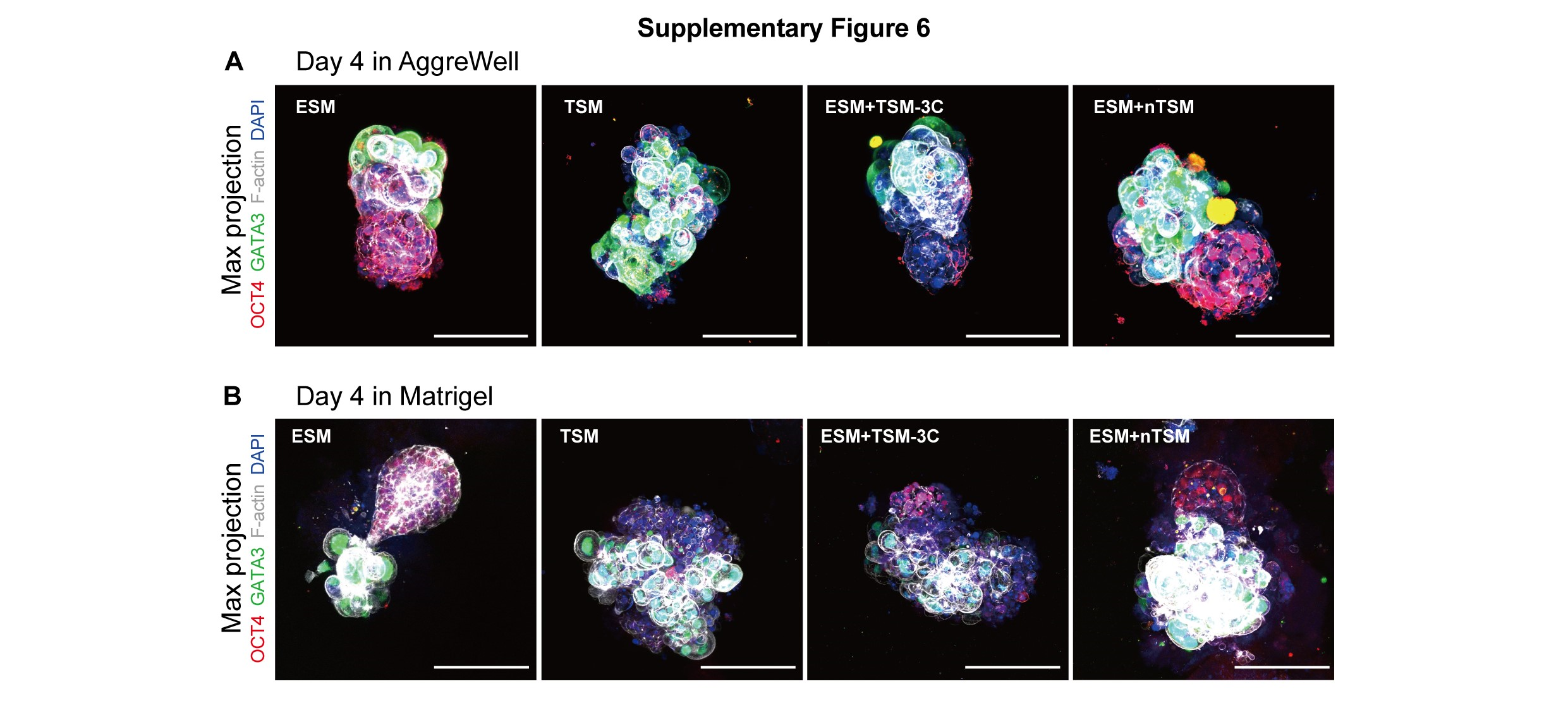


**Figure S6. ISRIB facilitates the organization of hESC and hTSC aggregates in 3D conditions. Related to Figure 7.**

(A) The representative max-projection images of the immunofluorescent staining for F-actin (a lumenogenesis marker protein), OCT4 and GATA3 in the ETAs cultured in indicated mediums. Nuclei were stained with DAPI. Scale bars: 100 μm.

(B) The representative max-projection images of the immunofluorescent staining for F-actin, OCT4 and GATA3 in the ETAs at day 4. Scale bars: 100 μm.

The indicated co-culture mediums included ESM, TSM, ESM+TSM-3C and ESM+nTSM. ESM+TSM-3C, remove A83-01, SB431542 and CHIR99021 in 1:1 mixture of ESM and TSM. ESM+nTSM, 1:1 mixture of ESM and nTSM.
